# A new split based searching for exact pattern matching for natural texts

**DOI:** 10.1371/journal.pone.0200912

**Published:** 2018-07-26

**Authors:** Saqib Hakak, Amirrudin Kamsin, Palaiahnakote Shivakumara, Mohd Yamani Idna Idris, Gulshan Amin Gilkar

**Affiliations:** 1 Faculty of Computer Science and Information Technology, University of Malaya, Kuala Lumpur; 2 College of Computer and Information technology, Shaqra university, Saudi Arabia; Victoria University, AUSTRALIA

## Abstract

Exact pattern matching algorithms are popular and used widely in several applications, such as molecular biology, text processing, image processing, web search engines, network intrusion detection systems and operating systems. The focus of these algorithms is to achieve time efficiency according to applications but not memory consumption. In this work, we propose a novel idea to achieve both time efficiency and memory consumption by splitting query string for searching in Corpus. For a given text, the proposed algorithm split the query pattern into two equal halves and considers the second (right) half as a query string for searching in Corpus. Once the match is found with second halves, the proposed algorithm applies brute force procedure to find remaining match by referring the location of right half. Experimental results on different **S1 Dataset**, namely Arabic, English, Chinese, Italian and French text databases show that the proposed algorithm outperforms the existing **S1 A**lgorithm in terms of time efficiency and memory consumption as the length of the query pattern increases.

## 1. Introduction

**Fig 1:**
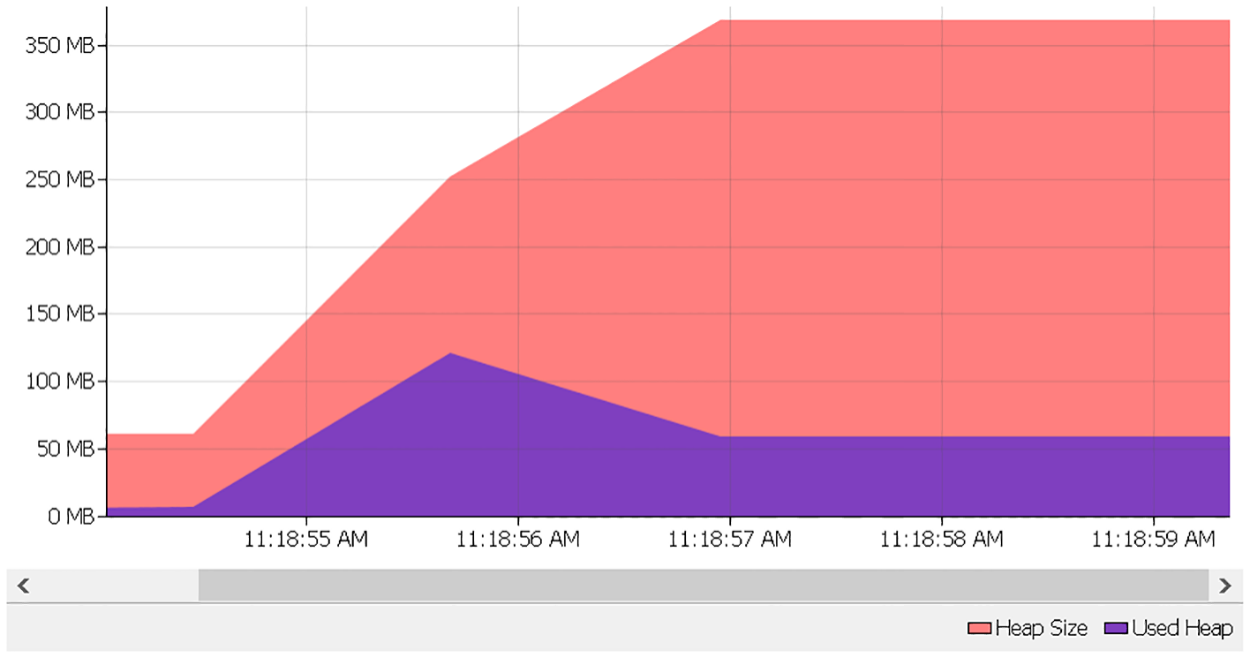
Memory usage of existing exact matching algorithms.

As swift changes in digital technologies, converting raw data to digital data and uploading to system online is also changing with the same proportionality. As a result, size of database increase drastically. Therefore, in order to cope with real-time applications and situation, there is a need for focussing on both time and space complexity of the systems or methods because these two parameters decide usefulness and effectiveness of the system despite the methods achieve good accuracy. Most of the existing methods in literature have focused on time complexity parameter and little attention has been paid towards space complexity (memory consumption) parameter. Therefore, there is a dearth of developing a method which achieves both times as well as space efficiency irrespective of the size of the database [1]. It is evident that in recent days, modern programming languages, such as Java and C# are widely used for setting up real-time systems because these software-based languages involve automatic memory management [2]. It is noted that heap size which is part of memory segment plays a major impact on the performance of garbage collection which in turn affects the overall performance of the systems having multiple processes [3]. For example, if heap size is less than the application requirement, it would cause excessive garbage collection while heap size more than the physical memory results in induce paging. On the other hand, there is no generalized criterion to decide the correct heap size according to application requirement [2]. This is beyond scope of this work. One such illustration using existing string matching [4] on Arabic dataset is shown in Fig 1 where we can see initially the algorithm requested 350 MB of the heap but it uses 70 MB (average) resulting in a waste of memory resources. Therefore, it is necessary to focus on both time and space complexities of the method.

**Fig 2:**
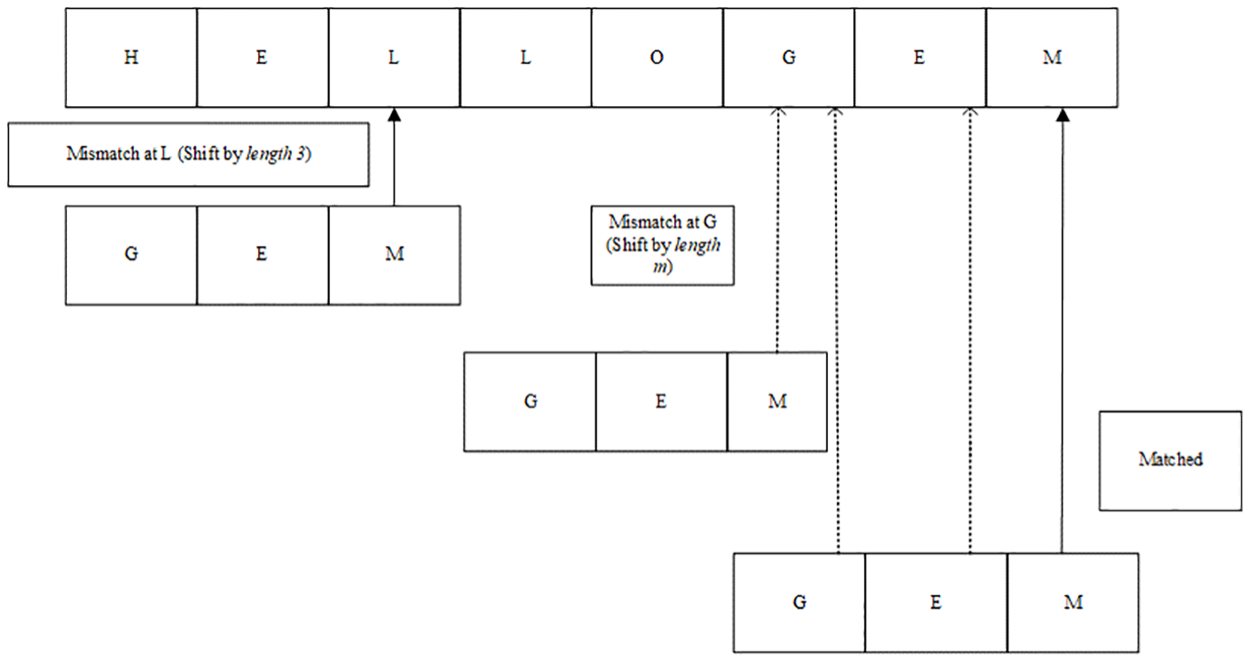
Boyer Moore algorithm [7].

The main reason for the existing exact string matching algorithms to consume more memory is the pre-processing involved in the computation of shifts. For example, in Fig 2, Boyer-Moore algorithm, [5] starts searching characters from right to left of the given query pattern. If there is a mismatch, algorithms shift as many as *m* characters according to the shift table computed in pre-processing phase. It looks similar to QS algorithm [6] with respect to finding a match, except BM algorithm uses both good suffix shift and bad-shift while QS algorithm uses only bad shift[7]. BM is one of the most standard and widely used algorithms in pattern matching and a lot of improvement in terms of time efficiency was carried out by post researchers to this very concept of character shifts. Few existing string matching algorithms using the same concept include fast search searching algorithm [8], modified Boyer Moore algorithm[9, 10], Horspool algorithm [11], Tuned BM [6], Turbo BM [12], SSM Algorithm [4] and so on [9, 10].

### 1.1. Motivation

The motivation of carrying out this research and proposing the idea was initially based on some experiments and observations. We found that most of the existing exact matching algorithms improve time complexity at the cost of memory wastage as shown in Fig 1. Besides, the advancement of the technologies like i-core 5/7 processors that can process bits much faster was another motivation. Our hypothesis assumed that the optimisation of brute force algorithm over fast i-core processors with more than 4 GM RAM will improve time and reduce heap memory wastage.

In Table 1, different input strings of varying lengths are given. The idea of splitting the string into two halves came from this very initial observation. Traditional exact matching algorithms need pre-processing to decide, how many letters to skip for the possible match. In case of splitting the given input into 2 halves and processing the right half first will improve the memory and time complexity was the core idea.

**Table 1:** Motivation to propose the idea of splitting.

Input String	Length of String (including white space)
He	2 characters
And the evening and the morning were the fourth days.	30–50 characters
And God said, Let the waters bring forth abundantly the moving creature that hath life, and fowl that may fly above the earth in the open firmament of heaven	More than 150 characters

## 2. Related work

One of the standard benchmark exact algorithms has been a Boyer-Moore algorithm (BM) as explained above. There are algorithms which proposed to overcome the drawback of the BM algorithm based on its good suffix and bad character rule. [11] simplifies the Boyer-Moore’s algorithm by removing the good suffix rule (Boyer-Moore-Smith Algorithm). [13] proposed algorithms which are an extension of BM algorithm focuses on computing the shift with the text character. Timo Raita (Raita, 1992) proposed algorithm known as Raita algorithm which is modified form of BM algorithm. [12] proposed Turbo-BM Algorithm which works based on dynamic simulation technique. Berry-Ravindran [14] proposed an algorithm, known as Berry and Ravindran algorithm which is an improvement over quick search algorithm. Ahmad [15] proposed an idea of exploring parallel processing for the two pointers that used in string matching process. i. e., one pointer starts searching from the left side and another pointer starts searching from the right side, thus it reduces overall search time. [16] proposed hashing technique to avoid a quadratic number of character comparisons [8]. However, the drawback of this approach is the possibility of hash collision. Similarly, there are bit-parallelism and automata-based exact matching approaches to improve the search time. The main issues with these approaches is that dependence on computer word size for matching and difficulty in implementation [8].

In the light of above discussion, it can be asserted that the primary focus of the existing method is time complexity [17], [18]. Researchers paid little attention towards space complexity (memory consumption), especially when database size increases continuously.

Thus, in this paper, we present a novel approach to solving the exact string matching problem which achieves both time and space efficiency. The main advantage of the proposed method is that it works well regardless of the type of database, unlike existing methods that depend on the type of the database. Besides, it is easy to parallelise this method and gain significant enhancement in decreasing time and memory requirements. This paper is organized as follows: Section 2 presents the proposed algorithm, Section 3 shows experimental results and Section 4 provides our conclusion.

## 3. Proposed algorithm

**Fig 3:**
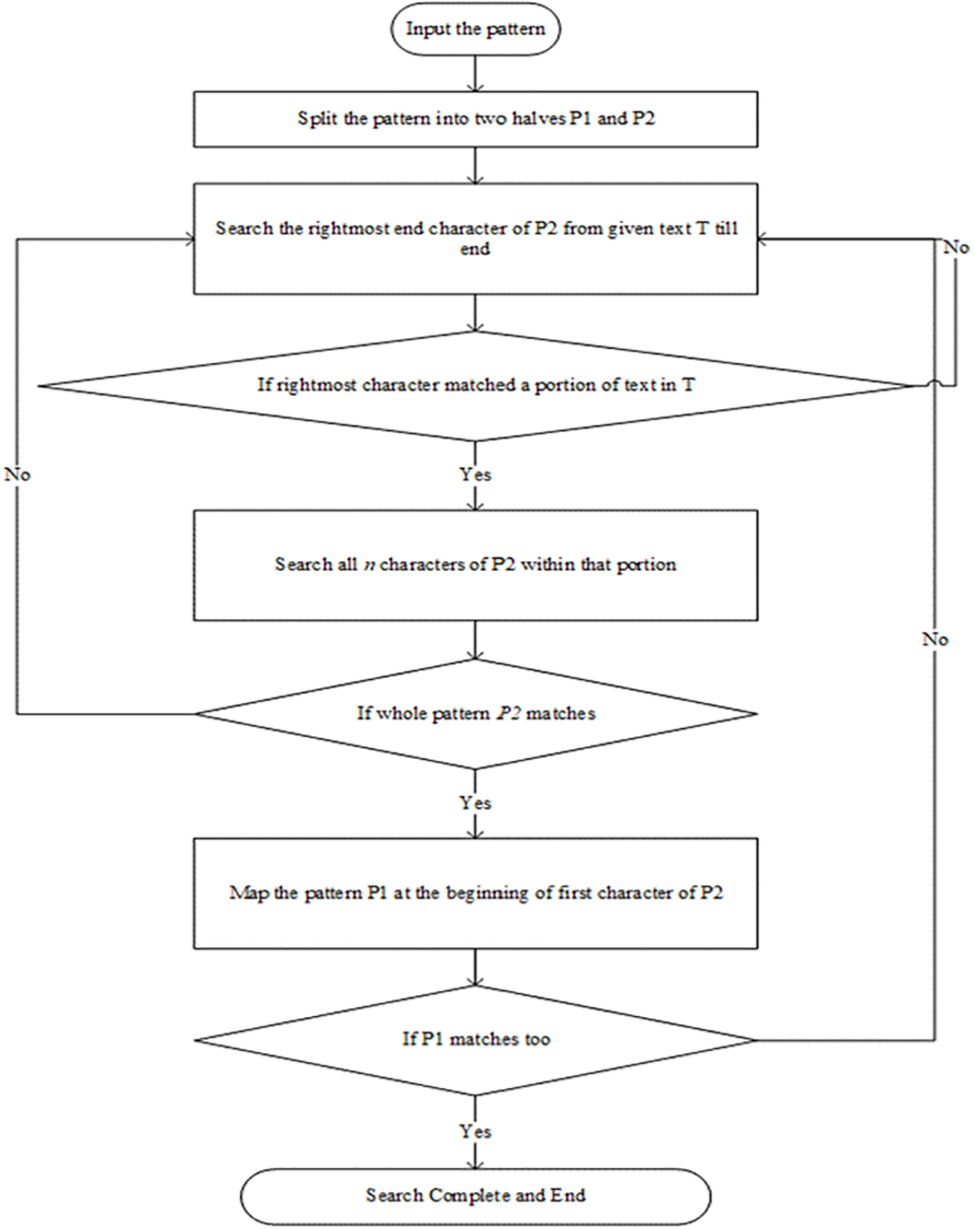
Flow chart of proposed algorithm.

As noted from the illustration shown in Fig 2 that since conventional exact string matching algorithms search query pattern at the character level, the algorithms consume more processing time and more space. This factor motivated us to propose an algorithm that uses a bunch of characters of query pattern for searching in the database. The proposed approach is based on the divide and conquer approach, where the pattern to be searched is split into two halves, say pattern *p* into *p1* and *p2* respectively. If string pattern length is even, it considers *p2* to find a match with the dataset. Once algorithms find a match, it matches *p1* with the adjacent characters found for *p2* directly. As a result, it saves lots of comparisons and memory consumption. The steps of the proposed algorithm can be seen in Fig 3. In this work, we determine the division of the given query string pattern into two equal halves empirically as shown in Table 1 where we conduct experiments for time and space complexity by varying size of the query string.

The algorithm more specifically is as follows. Suppose we need to find a *p* from given text *t* of length *n*. The proposed algorithm instead of considering the whole pattern *p* as an input, it searches *p*_*2*_ only such that (*m*_*2* < =_ *n*). Once right halve has found a match during searching process, the proposed algorithm considers the whole left half string to match with the database by considering reference found by right half string match. As a result, the proposed algorithm involves shift only for a right half in contrast to brute force algorithm or traditional exact matching algorithms where it involves a lot of shifts for a whole pattern. The proposed algorithm starts scanning from rightmost end to the leftmost end of the given text *t* and matching process of *p*_2_ starts from left to right i.e. (*i*_*0*,_*i*_*1*…._ *and*-1). In case, there is a match, *p*_*1*_ is mapped onto the location using the below-mentioned equation, where *i*_*0*_ denotes the position of rightmost character matched in *p*_*2*._

$$Position\;to\;Map\;(p_{map})\;=\;{(p}_{2}\text{[}i_{0}]-m_{2}\text{)}$$ (1)

**Fig 4:**
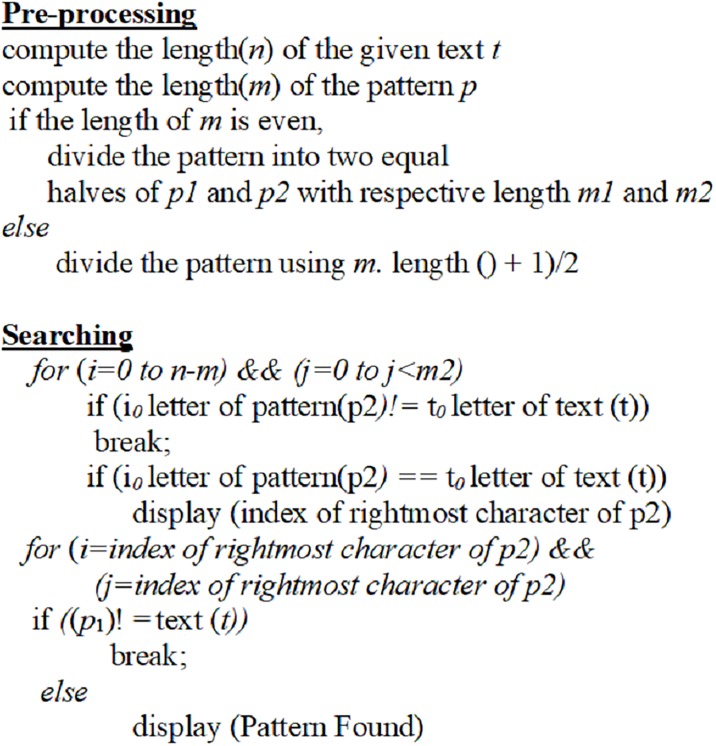
The pseudo code of proposed algorithm.

With the formula in Eq (1), if *p*_*1*_ also matches the given text, algorithms move to other location to verify the other matches. In case there is a mismatch, algorithm again starts scanning from the last matching position i.e. *i*_*0*._ The pseudo code of the proposed algorithm is presented in Fig 4.

**Fig 5:**
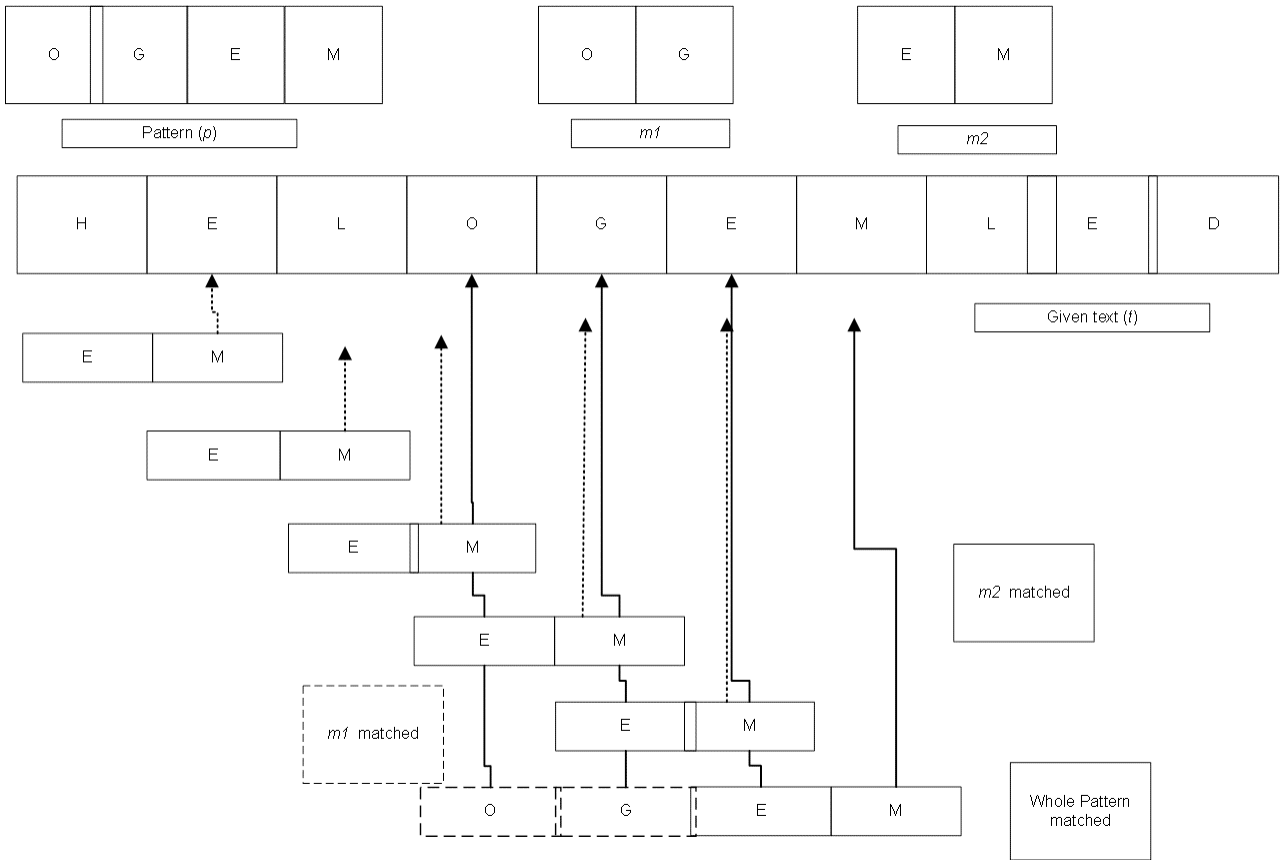
Working example of proposed algorithm for even length pattern.

**Fig 6:**
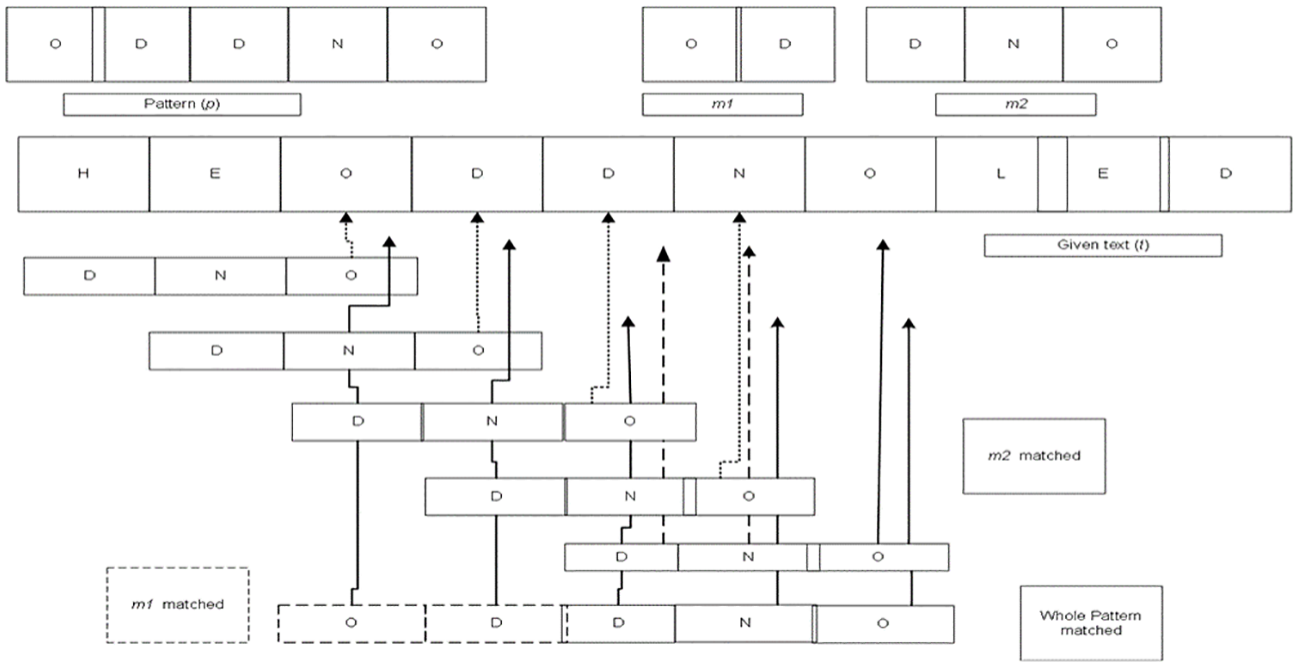
Working example of proposed algorithm for odd length pattern.

The proposed algorithm is illustrated in Figs 5 and 6 for a query string of even and odd lengths, respectively. Fig 5 shows that for a given text, (*t*) “HELOGEMLED”, the proposed algorithms divide a query string “OGEM” into “OG”, say, *p1* and “EM”, say *p2*. Since the query string length is even, it divides into two equal sub-strings. Then the proposed algorithm scans *p2* in *t* until it finds a match for “M”.

In the first search phase, “M” do not match with “E” of given text (*t*). Thus, the pointer will move to *i*_*0*_+1 position, again encountering mismatch with “L”, followed by “O”, “G” and “E” respectively. Finally, there is a match where character “M” of sub-pattern (*p2*) matches character “M” in the text (*t*). Now, the pointer moves to (*i*_*0*_-1) position of pattern *p2*_._ Finally, the next character of pattern i.e. “E” also matches the text, indicating the match. At last, *p1* is mapped directly based on the location of the last match.

In the same way, we illustrate the searching process of the proposed algorithm for the odd length of query string in Fig 6 where it considers sub-string which contains more characters as *p2* after division.

## 4. Experimental results

**Fig 7:**
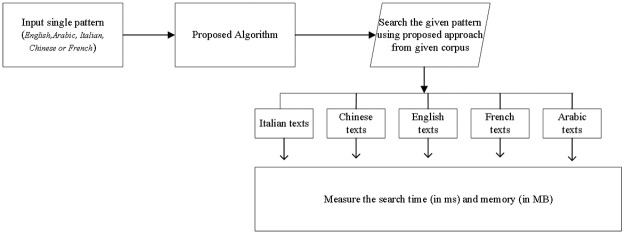
Experimental framework.

To evaluate the proposed algorithm, we consider a standard S1 Dataset of different scripts, namely, English, Italian, Chinese, French, and Arabic that Alsulami [4] has taken for comparison purposes from the work of Faro [10] and Tanzil.net[19]. It is noted that Arabic and Chinese database uses UTF encoding scheme because of diacritics and each character of these two scripts considers one-byte information while other datasets use ASCII encoding. The main reason to consider a dataset of the different script is to show that the proposed algorithm is script independent and takes less amount of memory for all scripts. The experimental framework is presented in Fig 7.

Since our objective is to evaluate the proposed method in terms of time and space-time complexity, we use processing time in milliseconds and memory consumption in MB as two performance measures. The same measures are used for all experimentation on different script S1 Dataset.

To show effectiveness and usefulness of the proposed algorithm, we compare the results of the proposed algorithm with the results of well-known S1 Algorithm on different datasets. The existing S1 Algorithm are Boyer-Moore (BM) algorithm which considers the rightmost character of the pattern for searching and it uses good-suffix shift and bad-character shift during matching. [6] proposed an algorithm which is called BMT because it is improved version of BM algorithm. This algorithm combines the strengths of BM and KMP (Knuth-Morris) algorithms. [12] proposed Turbo-BM (TBM) Algorithm which records the suffix of last matched sub string of the pattern with which it jumps over sub-string to allow execution of turbo-jump (memory match), unlike other algorithms. Recently, character-based approach by [4] proposed algorithm (SSM) which compares the pivot character with the corresponding character and shifts the pattern either using Horspool shift or hybrid shift. In the work of Faro [10], Hash 3 and SBNDM algorithms have shown better results among 85 algorithms for natural texts like the bible. For that reason, SSM algorithm [4] has been compared with Hash 3 and SBNDM algorithms. SSM algorithm has shown better results than hash 3 and SBNDM indicating that the results are better than those existing 85 algorithms. It is observed from the review of existing methods that all four existing methods use character components for matching and searching. Thus, it involves more number of computations, comparisons, and shifts which results in more time processing and memory consumption. Furthermore, for the all the above existing methods, traditional brute force criterion is common. Therefore, we also use the same criterion for comparative study without additional features in this work.

For experimentation, we consider short query pattern (1–4 characters’ length), medium query pattern (5–8 characters length) and long query pattern (more than 8-character length) to test time and space efficiency of the proposed and existing S1 Algorithm on different S1 Dataset. Each algorithm is run 10 times and running times were calculated by taking mean of 10 running times. The implementation is done using the NetBeans 8.02 on i-5 Intel Processor with 4 MB caches, 4 GB RAM using Windows 10.

### 4.1 Outcome of experiments

The quantitative results of the proposed and existing S1 Algorithm for query pattern lengths on different S1 Dataset are reported in Tables 2–4 where we can notice that the proposed algorithm outperforms existing S1 Algorithm for all the queries except TBM on Arabic, French text, BMT on the Italian and French text with SSM on Italian text. Therefore, it can argue that the proposed algorithms are effective in terms of time efficiency for most of the S1 Dataset. Since the aim of the proposed algorithm is to achieve better time efficiency, it reports bit poor results for Italian and French texts. The reason for poor results is probably due to the nature of these datasets with respect to the arrangement of selected patterns. This is especially worse for very short patterns of size <4. However, in case of medium patterns i.e. pattern length 4–7, on an average SSM algorithm performed much better as this algorithm performs best under this scenario due to its dynamic pivot pointer that involves a maximal safe shift in case of mismatch on rightmost end of the pattern. For longer patterns again, on an average proposed algorithm performed better compared to existing S1 Algorithm.

**Table 2:** Processing time in milliseconds of the proposed and existing S1 Algorithm for the query pattern length of fewer than four characters (short).

Corpus	Text	Size (MB)	Sample patterns	BM	BMT	TBM	SSM	Brute Force	Proposed
Arabic	Quran	0.7	بِسْمِ	1062	985	939	1235	2152	937
English	Bible	3.83	Good, the	5967	5396	6027	5710	8527	4205
Italian	Orlando	0.72	Dal, tal	5172	4058	5060	4230	13430	4492
Chinese	Journey	1.37	兒	3025	3969	3158	2806	7547	2500
French	L’homme	1.13	Dans, ils	3078	2284	2249	2859	6995	2549

**Table 3:** Processing time in milliseconds of the proposed and existing S1 Algorithm for the query pattern length of 4–7 characters (medium).

Corpus	Text	Size (MB)	Sample patterns	BM	BMT	TBM	SSM	Brute Force	Proposed
Arabic	Quran	0.7	الرَّحِيمِ	969	1000	938	906	2050	878
English	Bible	3.83	Finishe, wroth	2390	2302	2281	2260	7547	2797
Italian	Orlando	0.72	Tratto, saetta	3843	4112	4177	3474	13310	4391
Chinese	Journey	1.37	隔開	2313	2047	2318	2297	7371	2578
French	L’homme	1.13	C’est, epoque	2847	2288	2179	2531	7476	2400

**Table 4:** Processing time in milliseconds of the proposed and existing S1 Algorithm for the query pattern length of more than 8 characters (long).

Corpus	Text	Size (MB)	Sample patterns	BM	BMT	TBM	SSM	Brute Force	Proposed
Arabic	Quran	0.7	بِسْمِ اللَّهِ الرَّحْمَنِ	1094	1013	1031	1078	2100	844
English	Bible	3.83	Continually, that Adam	2172	2253	2157	2422	7939	2092
Italian	Orlando	0.72	Trascorso, lungo tratto	4143	3937	3920	3330	13648	4375
Chinese	Journey	1.37	旗飛彩	2083	2093	2185	2218	7655	2609
French	L’homme	1.13	Angleterre, imitele chinois	2869	2374	2337	2719	7105	2384

**Fig 8:**
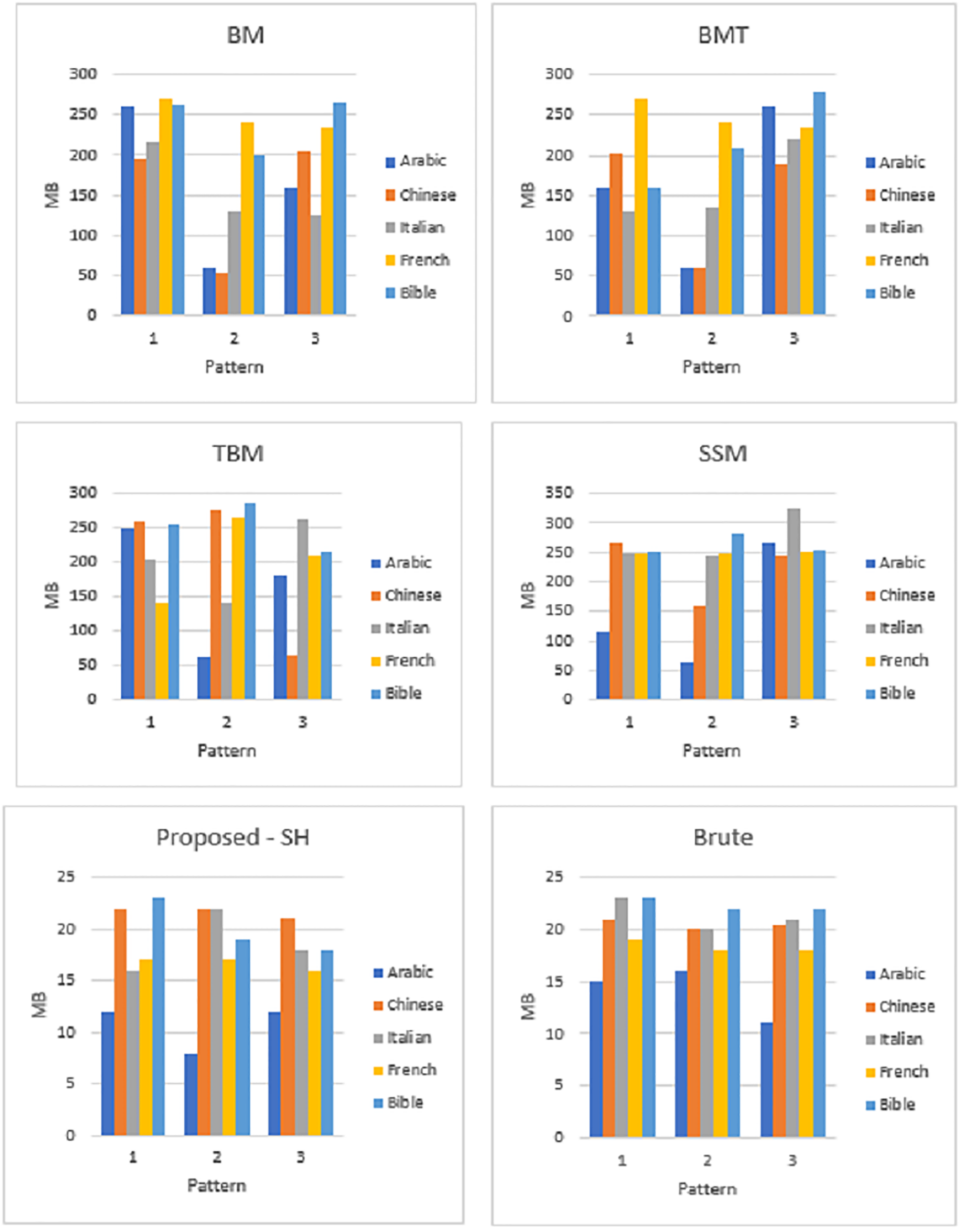
Memory analysis.

**Fig 9:**
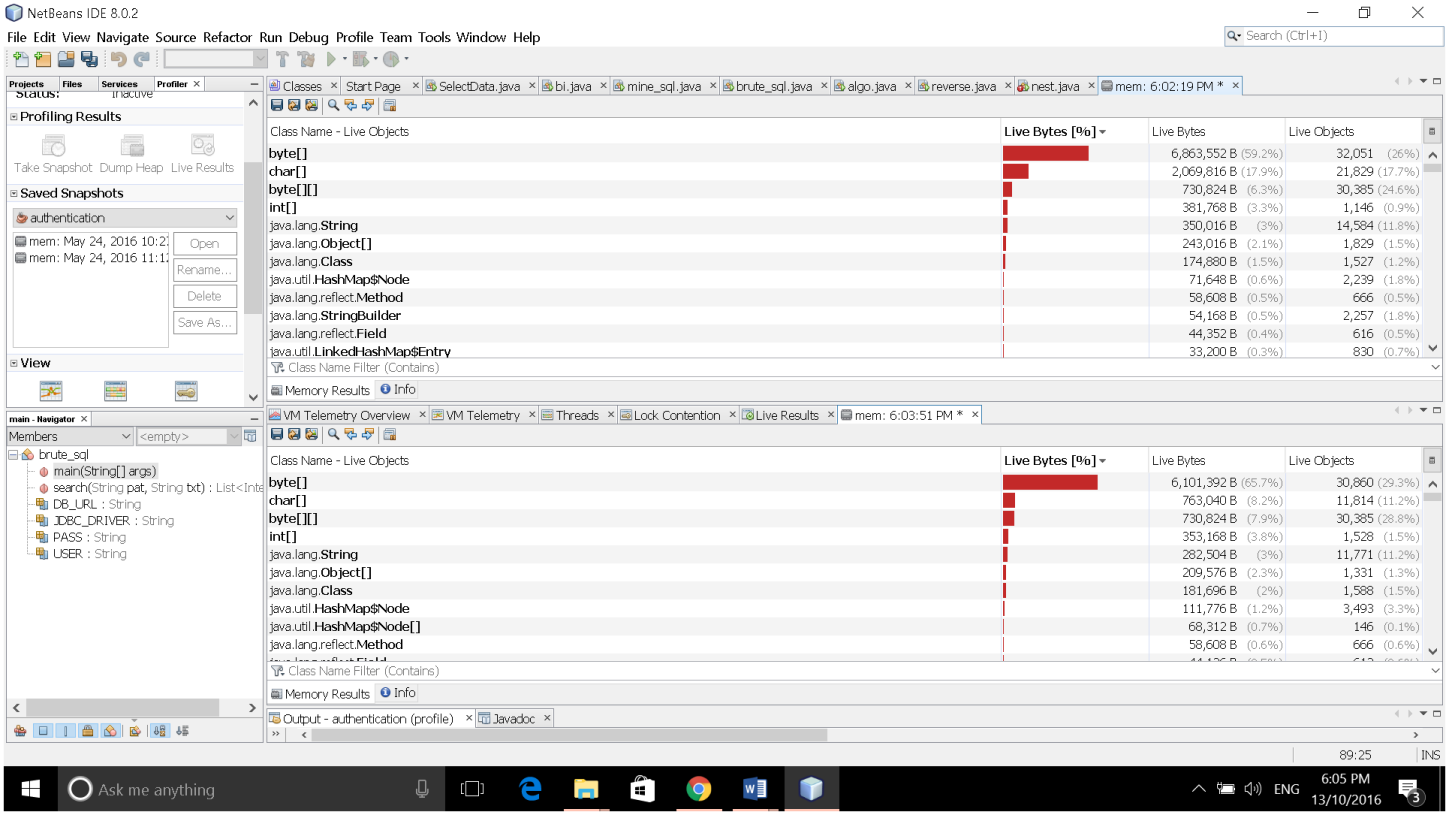
Brute force memory analysis.

In the same way of experiments on time efficiency for the same S1 Dataset, we calculate memory used for matching and searching on the query words listed in Tables 2–4. The average of memory consumption of different query words of the proposed and existing S1 Algorithm on different S1 Dataset are shown in Fig 8. Fig 8 shows that all the existing S1 Algorithm except Brute force and proposed algorithm consume 100 to 200 MB of heap memory during runtime. However, Brute force algorithm consumes little memory i.e. less than 20 MB. It requires more time for searching according to Tables 2–4. Brute force algorithms require more operations for searching string in the database. Since it requires more operations, usage of pointers, calling internal methods and variable also increases. Therefore, brute force algorithms consume more space than the proposed algorithm. For the fair comparative study, we use the function in Java for estimating memory consumption for all the experiments shown in Fig 9. Memory requirements were analysed using memory analysis tool available in java using NetBeans IDE [20]. From Fig 9, it can be seen upper portion is memory requirements of the proposed algorithm and lower part of brute force algorithm. Although there is not much difference in terms of memory requirements (ranging from 15–25 MB) between the two. But, the slight difference with respect to memory requirements depends on the allocation of bytes and char inside JVM that take some amount of memory. Therefore, we can confirm that the proposed algorithm achieves both time and space efficiency for different query pattern length on different S1 Dataset.

## 5. Conclusion and future work

In this paper, we have proposed a novel idea for exact string matching to achieve both time and space efficiency regardless of query pattern length, dataset size and scripts. The proposed algorithm split given query pattern length into two halves and then it considers right halve for searching in a text. Once the match is found for right halve, the proposed algorithm uses left halve directly from the matched reference. This process helps in reducing the number of computation especially comparisons at the same time it consumes less memory due to no pre-processing involved as compared to existing exact matching algorithms. To show the usefulness of the proposed method, we have conducted experiments on datasets of different S1 Dataset scripts, namely, Arabic, English, and Chinese. Experimental results of the proposed and existing S1 Algorithm on different S1 Dataset for different query pattern length show that the proposed algorithm outperforms most of the existing S1 Algorithm in terms of time and space efficiency. Therefore, we can assert that the proposed algorithm is a script, query pattern length and dataset size independent.

In near future, we are planning to extend the proposed algorithm to solve string matching with multiple string matching and approximate string matching. To handle scalability on huge databases with different scripts, formats etc, we are investigating to introduce keyword spotting for exact matching such that the algorithms spot the word, which represents the whole page. In this case, no need to scan the whole page from beginning to end of the page. In addition, the approach considers semantics layout of the page for searching keywords of the page. It is valid because any book or number pages in the book have some logical flow at the semantic level. The new approach is to explore such high-level semantics to reduce search time, at the same time, it should extract right information with minimum memory usage.

## Supporting information

S1 Dataset Benchmark datasets. (RAR)

S1 Algorithm Algorithm-sources. (JAR)
